# What Types of Greenspaces Are Associated with Depression in Urban and Rural Older Adults? A Multilevel Cross-Sectional Study from JAGES

**DOI:** 10.3390/ijerph17249276

**Published:** 2020-12-11

**Authors:** Miho Nishigaki, Masamichi Hanazato, Chie Koga, Katsunori Kondo

**Affiliations:** 1 Graduate School of Medical and Pharmaceutical Sciences, Chiba University, 1-8-1 Inohana, Chuo-ku, Chiba-shi, Chiba 260-8672, Japan; 2 Center for Preventive Medical Sciences, Chiba University, 1-33 Yayoi-cyo, Inage-ku, Chiba-shi, Chiba 263-8522, Japan; hanazato@chiba-u.jp (M.H.); chiekoga@chiba-u.jp (C.K.); kkondo@kkondo.net (K.K.); 3 Center for Gerontology and Social Science, National Center for Geriatrics and Gerontology, 7-430 Morikoka-cho, Obu-shi, Aichi 474-8511, Japan

**Keywords:** older adults, depression, mental health, greenspace, trees, grasslands, fields, built environment, urban, rural

## Abstract

Depression in older adults is a public health challenge. We aimed to clarify the relationship between depression in older adults and three types of neighborhood greenspaces: trees, grasslands, and fields. We utilized data from the Japan Gerontological Evaluation Study (JAGES) performed in 2016. Multilevel logistic regression analysis was used for non-stratified and stratified analyses for the urban–rural regions. The target population comprised 126,878 older adults (age ≥ 65 years) who responded to the depression questions and were living in 881 neighborhoods in Japan. Depression was diagnosed based on a Geriatric Depression Scale score ≥5, and 20.4% of the study population had depression. In the pre-stratification analysis, areas with more greenspaces revealed lower odds of depression (odds ratio (OR) 0.95, 95% confidence interval (CI) 0.85–0.95). In urban areas, more trees correlated with lower odds of depression (OR 0.94, 95% CI 0.89–1.00). In rural areas, moderate amounts of grassland were associated with lower odds of depression compared to areas with fewer grasslands (OR 0.91, 95% CI 0.83–1.00). We found that urban areas with higher tree density and rural areas with moderate amounts of grassland were associated with lower odds of depression.

## 1. Introduction

Depression is considered one of the causes of deterioration in physical and cognitive functions and premature death. In older adults, it is an important public health challenge [1]. Among all age groups, the rate of depression is highest in older adults [1]. Reports indicate that mental health improvements are related to the number of greenspaces in an area [1,2,3,4,5,6,7]. For example, sufficient greenspaces in a region can increase physical activity, social contact, and biodiversity, reduce stress, improve exposure to better air quality, and mitigate the heat island effect, which are related to better mental health status [2].

Several studies have focused on the association between greenspace and health; however, research on the types of greenspaces and their associations with older adults’ depression is limited. A study assessing the difference between trees and grasslands in urban areas found that a large number of trees increased the subjective health of adults, while the same was not observed for the grasslands [8]. Having trees in urban areas was also associated with more walking by adults [9]. Moreover, there was a reduction in the rate of depression among older adults living in nursing homes situated in neighborhoods that had more trees [10]. In a longitudinal analysis, a large number of grasslands in rural areas was associated with good mental health in older adults [11]. These findings indicate that the impact of greenspace on depression among older adults may vary depending on the type of greenspace and urban and rural characteristics.

Many of these previous studies assessed the relationship between greenspace and mental health in urban populations only [4,6,7]. Regional differences, such as urban and rural areas, may have different effects on the relationship between the type of greenspace and the health of older adults [11]. For example, considering exposure to trees, for those in urban areas, street and park trees are easy to access. However, trees in rural areas are often located in places that are difficult to access, like the forests, which makes exposure to trees difficult. Unlike urban areas, grasslands and fields occupy large areas in rural regions. Thus, greenspace factors related to depression in older adults may differ between urban and rural regions.

Urban and rural perspectives play an important role in social gerontology [12,13]. Therefore, it is necessary to study the relationship between the local environment and the health of older adults in both urban and rural regions. A study on urban and rural differences in China found that older adults in rural areas had higher levels of depression than those in urban areas [14]. According to a study comparing urban and rural Japan, older adults in rural areas display lower social participation and higher dementia risk than those in urban areas [15,16]. Moreover, depression is one of the factors of dementia in older adults [1]. In this regard, it is necessary to clarify the association between urban and rural regions and their respective greenspaces with depression in older adults. To the best of our knowledge, no studies have assessed the association between the types of urban and rural greenspaces and depression in older adults who do not require long-term care or assistance.

Therefore, the first objective of this study was to clarify the relationship between depression and the prevalent types of greenspaces (trees, grasslands, and fields) in older adults in Japan. Next, we clarified whether urban and rural differences in residential areas influenced the relationship between depression and the type of greenspace.

## 2. Materials and Methods

### 2.1. Selection and Description

#### 2.1.1. Description of the Setting

**Figure 1:**
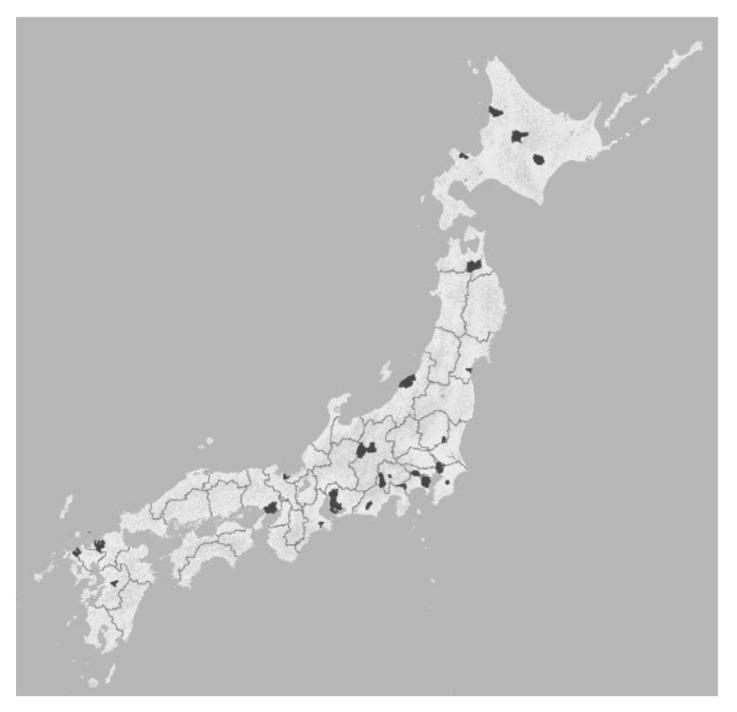
Study area.

We used cross-sectional data from the Japan Gerontological Evaluation Study (JAGES) 2016 [17,18]. This is a survey of independent men and women over the age of 65 years, not in long-term care. The survey areas comprised 39 municipalities in 18 prefectures out of 47 prefectures in Japan. (Figure 1). The municipalities include urban/suburban and rural communities from the north (Hokkaido; the northernmost area) and south (Kyushu; the south area) in Japan. The survey was conducted between October 2016 and January 2017, and questionnaires were mailed to the potential participants. The survey participants were extracted on a municipal basis, and the sampling frame was based on the information of the long-term care insurance certification data for the older adults aged 65 years and above, excluding those requiring long-term care, depending on the size and budget of the municipality. Questionnaires were distributed to all participants when the number was less than 5000 and to those selected by random sampling when the number was 5000 or more. Of the 279,661 questionnaires distributed, 196,091 (effective collection rate, 70.1%) were completed. Although the JAGES respondents were not randomly selected, the data covered a wide range and size of community populations. This research protocol and informed consent method were approved by the Ethics Committee of Nihon Fukushi University (No. 13–14).

#### 2.1.2. Selection and Description of the Setting

We used the school district as the neighborhood unit. In Japan, it is common for municipalities to specify elementary schools and junior high schools according to the student’s address. In other words, the range of municipalities is divided by multiple school districts. Therefore, this school district was used as a neighborhood unit. School districts can be used as venues for local festivals, polling stations for elections, evacuation shelters in the event of a disaster, etc., and can be used as a unit to define the scope of the local community. The school district represents a geographical area that is easy for seniors to navigate [19]. The average size of a school district was 8.57 km^2^ (SD = 27.49).

### 2.2. Outcome Variables

The Geriatric Depression Scale (GDS) [20,21] with its 15 items (score range: 0–15 points) were used to determine the presence of depression. Those with a total GDS score of 5 or more were considered to have depression or a tendency towards depression [22,23].

### 2.3. Types of Greenspace

**Figure 2:**
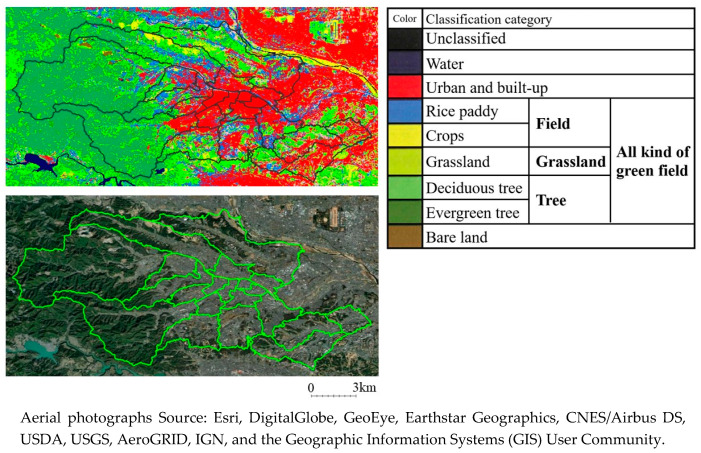
Example of High-Resolution Land-Use and Land-Cover Map (HRM) data and aerial photography of the target area (Hachiouji-city, Tokyo).

Using Geographic Information Systems (GIS), all types of greenspaces and a percentage of the trees, grasslands, and fields were intersected by the school district. This greenspace data were created based on the High-Resolution Land-Use and Land-Cover Map (HRM) products by the Japan Aerospace Exploration Agency (JAXA) and the Earth Observation Research Center (EORC) [24,25]. The resolution during HRM was 10 × 10 m. The HRM data classifies areas into 10 types of land use: water, urban and built-up, rice paddy, crops, grassland, deciduous tree, evergreen tree, bare land, unclassified, and no data. In this study, we defined a tree as a covering of a deciduous and evergreen tree. The field comprises a combination of rice paddy and crops. However, we did not change the definition of grassland. All kinds of green field were defined as a combination of trees, grassland, and field. For this analysis, we calculated the ratio of the area of greenspace in each school district to the area of the school district. We divided the proportion of each greenspace area into tertiles. Figure 2 shows an example of HRM data and aerial photographs of the target area. It shows the target areas, which included a city in Tokyo, the capital of Japan, and 20 school districts. It also illustrates the urban neighborhoods with most trees.

Aerial photographs Source: Esri, DigitalGlobe, GeoEye, Earthstar Geographics, CNES/Airbus DS, USDA, USGS, AeroGRID, IGN, and the Geographic Information Systems (GIS) User Community.

### 2.4. Individual-Level Covariates

The covariates for individuals included sex (male and female), age (65–69, 70–74, 75–79, 80–84, and ≥85 years), educational attainment (≤9 years, ≥10 years, or missing), annual household income (<2 million Yen, 2–4 million Yen, >4 million Yen, or missing), family composition (single-living, 2 or more living, others, missing), employment (working, retired and not working now, never had a job, missing), frequency of going out (<4 times a week, ≥4 times a week, or missing), whether they drive by themselves, the occupation with the longest duration of employment (other than agriculture, agriculture forestry and fisheries, forestry and fisheries, never had a job, or missing), and residence years (<10 years and ≥10 years). Being able to drive a car by oneself, and the occupation with the longest duration of employment being agriculture forestry, were considered covariates because mparticipants with these factors were more likely to be exposed to greenspace regardless of the neighboring environment. Other covariates were included based on previous studies [22,26]. Unknown data in the dataset were categorized as “missing” in this analysis.

### 2.5. Neighborhood-Level Covariates

Annual total daylight hours, deepest annual snowfall amount, and annual rainfall were included as regional covariates. Daylight hours were covariates because they are associated with seasonal depression and green growth [27] and are related to physical activity, which interacts with snowfall and annual rainfall, as well as green growth [28]. The abovementioned covariates were surveyed in 2010 by the Ministry of Land, Infrastructure, Transport, and Tourism (MLIT) in Japan, and they used 1 km of mesh data issued in 2012 [29]. The average value of the specified regional covariates for each school district was calculated using GIS. Additionally, we conducted a pre-stratification analysis that considered the population density of the residential areas. Residential areas were defined as building sites in the land-use from the MLIT. The resolution was 100 × 100 m [30]. The population density of the residential areas was calculated by equally distributing the population in the 500 m mesh data from the census of the building site using GIS, and by equally distributing the population by the neighborhood district polygon.

### 2.6. Statical Analysis

We conducted a descriptive analysis to summarize the participants’ characteristics. We also performed correlation analysis to explore the association between neighborhood-level covariates. The mean, standard deviation (SD), and minimum and maximum values of each variable at the neighborhood level are shown, along with the results of the Spearman’s ρ test. We then performed a multi-level logistic regression analysis using random intercepts and a fixed slope to investigate the association between neighborhood-level greenspace and individual-level depression using a non-stratified sample, in addition to the stratified analysis by urban and rural characteristics. The pre-stratification analysis included a total of 126,878 respondents (level 1) nested within the 881 neighborhood districts (level 2). The analysis conducted for urban areas included a total of 93,055 respondents (level 1) nested within 710 neighborhood districts (level 2). The analysis conducted for the rural areas included a total of 33,823 respondents (level 1) nested within 171 neighborhood districts (level 2). The odds ratio (OR) of depression and 95% confidence intervals (CI) were calculated.

The urban area referred to here, a Large Metropolitan City in an Organization for Economic Co-operation and Development (OECD) Functional Urban Area (FUA) [31], has a population of more than 1.5 million. This area is part of a Large Metropolitan City. The rural areas are not covered by the FUA’s Large Metropolitan City since their population is less than 1.5 million. The OECD FUAs are considered as internationally comparable cities defined and adopted in 39 countries. The urban and rural regions were analyzed in two models with different explanatory variables. In Model 1, the explanatory variables include all greenspace types. In Model 2, the explanatory variables included trees, grasslands, and fields. Stata MP 14 (Stata Corp, College Station, TX, USA) was used to conduct the statistical analyses.

## 3. Results

### 3.1. Participants’ Characteristics

**Figure 3:**
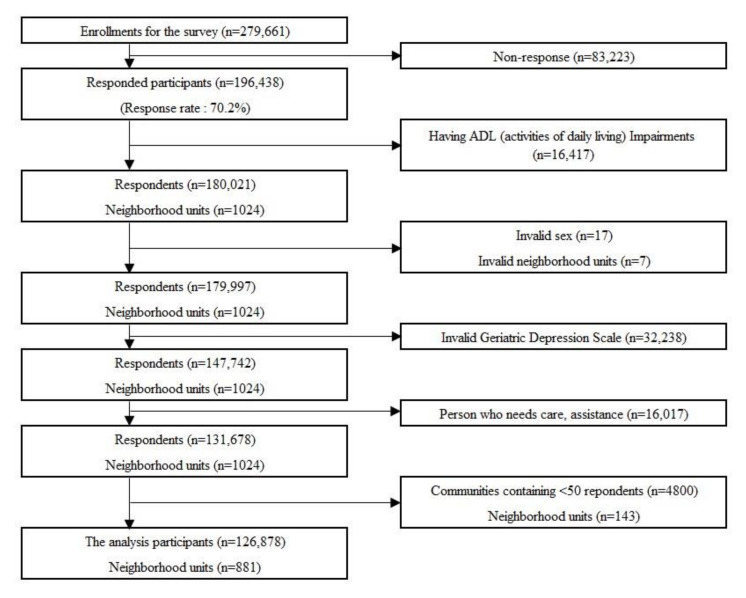
Flow chart of the survey participants.

This study targeted 881 neighborhood districts where individuals were physically and cognitively independent (i.e., not eligible to receive any benefit from public long-term care insurance), no data on sex, age, residential area, and depression responses were missing, and there were 50 or more respondents in a district. We analyzed the data from 126,878 subjects (Figure 3). The average area for the neighborhood districts was 9.6 km^2^ (standard deviation [SD] = 5.2 km^2^).

Table 1 shows the characteristics of participants with depression. The prevalence of depression was 20.4% (*n* = 25,846). Women constituted 51.5% of the participants, and more than half were under 74 years old. The proportion of participants with an educational attainment of 10 years or more was 69.7%. The largest proportion based on annual household income was <20,000 dollars (38.5%), followed 20,000–39,999 dollars (33.3%). The average income in 2014 for older adults households announced by the Japanese government (households comprising only persons aged 65 years and above or households in which unmarried persons under the age of 18 are added) was 21,160 dollars, assuming a rate of 100 yen per US dollar [32]. We found that 70.9% of the participants lived with others, and 26.1% are still working. The average weekly outing frequency was 74.9% for those who went out more than four times a week. As one of the means of transportation, 58.7% of the participants drive a car. The type of occupation with the longest duration of employment was agriculture, forestry, and fisheries (AG). The proportion of participants likely to be exposed to greenspace and bluespace by occupation was 3.5%. We also showed that 90.4% of the participants have lived in their current residence for more than 10 years.

### 3.2. Results of the Neighborhood Level Correlation Analysis

Table 2 shows the Spearman’s correlation coefficients for the neighborhood-level measures. All greenspace types were correlated with trees, fields, and grasslands (ρ = 0.78, 0.77, 0.70, respectively). All greenspace types (AG) were inversely correlated with residential population density (ρ = −0.69). The co-efficient of trees and grasslands was 0.51, while that of fields and grasslands was 0.52. Our analysis showed that trees, fields, and grasslands were associated. The population density of residential areas was strongly inversely associated with AG, fields, and grasslands (−0.688, −0.623, −0.537) and weakly associated with trees (−0.345). This suggests there are areas with many trees, even in urban areas.

### 3.3. Results of the Pre-Stratification Analysis

**Figure 4:**
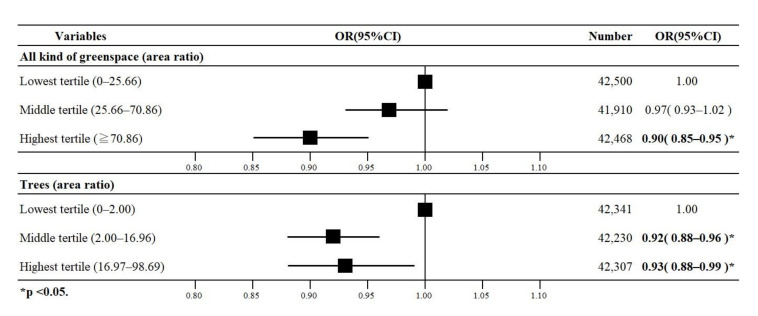
Odds ratios (ORs) with 95% confidence intervals (95% CI) for the association of all kinds of greenspace and trees with depression among older Japanese adults (*n* = 126,878).

The main results of multi-level logistic regression analysis for the pre-stratification are shown in Figure 4, and the details are shown in Table 3. According to Model 1 in Table 3, the highest tertile of all the greenspace areas had a lower oddof depression than their respective reference groups (OR 0.90, 95% CI 0.85–0.95). According to Model 2, the highest and middle tertiles of trees had lower odds of depression than their respective reference groups (OR 0.93, 95% CI 0.88–0.99; and OR 0.92, 95% CI 0.88–0.96, respectively). No statistically significant associations were observed between grasslands, fields, and depression.

### 3.4. Results of the Urban Area Analysis

**Figure 5:**
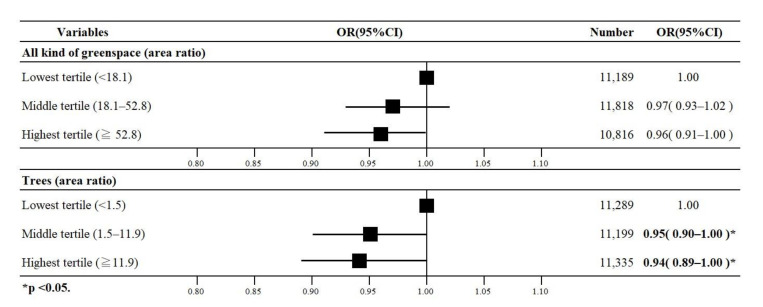
Odds ratios (ORs) with 95% confidence intervals (95% CI) for the association of all kinds of greenspace and trees with depression among older adults in urban areas (*n* = 93,055).

The main results of multi-level logistic regression analysis for the pre-stratification are shown in Figure 5, and the details are shown in Table 4. Table 4 shows the results of the multi-level logistic regression analysis, stratified by urban and rural. In urban areas, 18,474 (19.9%) people had depressive symptoms. In Model 1, the highest tertile of all greenspace areas had a lower oddof depression than their respective reference group (OR 0.96, 95% CI 0.91–1.00). In Model 2, the highest and middle tertiles for trees showed lower odds of depression than their respective reference group (OR 0.94, 95% CI 0.89–1.00; and OR 0.95, 95% CI 0.90–1.00, respectively). No statistically significant associations were observed between grasslands, fields, and depression.

### 3.5. Results of the Rural Area Analysis

**Figure 6:**
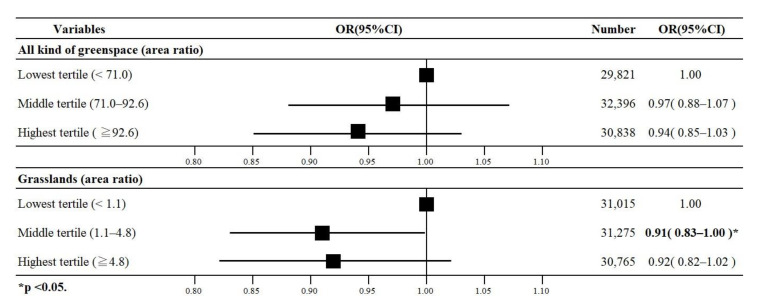
Odds ratios (ORs) with 95% confidence intervals (95% CI) for the association of all kinds of greenspace and grasslands with depression among older adults in rural areas (*n* = 33,823).

The main results of multi-level logistic regression analysis for the pre-stratification are shown in Figure 6, and the details are shown in Table 4. As shown in Table 4, in rural areas, 7372 (21.8%) people were reported to have depressive symptoms. In Model 1, no statistically significant association was observed between all types of greenspaces and depression. According to Model 2, the middle tertile of grasslands had a lower oddof depression than their respective reference group (OR 0.91, 95% CI 0.83–1.00). No statistically significant associations were observed between trees, fields, and depression.

## 4. Discussion

To the best of our knowledge, this is the first study to demonstrate the contextual relationship between types of neighborhood greenspaces and depression in independent older adults. This study also adds to the evidence reported by previous studies by revealing that the association between the types of greenspaces and depression differ between urban and rural areas. The results of this study are supported by the relationship of greenspaces with good mental health reported in previous studies [3,5]. With regard to the difference in the types of greenspaces, we found that more trees were associated with lower odds of depression; however, this was not the case for grasslands and fields

### 4.1. Results from the Urban Stratified Analysis

In urban areas, higher rate of tree areas was associated with lower odds of depression. This finding is similar to previous research on street-view green [33] and urban green types [8] of street-view. Urban trees are considered to have a superior effect in reducing the heat island phenomenon, mitigating noise, and improving the air quality compared to grasslands and fields [3]. This is because the trees have more greenness than the grasslands and fields in the same area; hence, the green effect may be greater. It is also thought that trees are more resistant to changes in weather and seasons than plants in fields and grasslands and have a positive effect on the environment over a long period. These positive effects of trees on the neighborhood are likely to encourage older adults to go out and reduce stress. It is known that having more trees in urban areas is related to the length of walking distance of older adults [9] and physical activities such as walking are associated with a lower rate of depression [2].

Urban trees improve subjective health [8]. Furthermore, greenspaces in an artificial environment may attract people’s attention and enhance their impression according to the Attention Restoration Theory [34,35]. Therefore, in urban areas, the presence of trees may have influenced the reduction in the odds of depression through the effects of greenspace on stress. For the Japanese people, *Shinrin Yoku* (forest bathing) is an ancient cultural tradition [36]. It has long been said that bathing in the power of trees is good for health. It has been reported that exposure to a magnificent view of nature, which people in urban areas lack, and nature therapy (e.g., forest bathing) are beneficial for the mental health of those who live in urban areas and are not engaged in occupations associated with agriculture, forestry, or fisheries [37]. According to the attention restoration theory, moving from an environment with fewer greenspaces to one with more greenspaces may gradually amplify the positive effects of greenspace on mental health.

### 4.2. Results from the Rural Stratified Analysis

In rural areas, all types of greenspaces and trees were not significantly associated with depression. These findings are similar to those from a previous cross-sectional study, in which the types of greenspaces were not associated with the subjective health in rural areas [38]. In contrast, a moderate rate of grassland areas was associated with lower odds of depression. Grasslands show a wider field of view, easier access, and cause less anxiety than trees. They may also be used as places for physical activity and socialization. Opening the field of vision to natural sights (e.g., landscapes and views) is related to social cohesion and mutual cooperation; thus, it is likely related to a lower incidence of depression [6].

Older adults in areas with more grassland may not show association with depression because they had less visibility and access to grasslands than in areas with moderate amounts, making them less accessible for physical activity and socialization. A previous study assessing the relationship between tree density and stress reported that moderate amounts of trees contribute to stress recovery, while large amounts of trees weaken stress recovery. For men, the dose–response curve was an inverted-U shape as tree cover density increased [39]. In the suburbs, there are too many trees in a neighborhood, which delays stress relief and may not be associated with less depression. Similar effects can be expected in areas with a lot of grasslands.

### 4.3. Features of the Analysis Method Used in This Study

This study included different methodological features compared to previous studies. First, we focused on the types of greenspaces. Several studies that analyzed the relationship between greenspace and health quantified the greenspace from satellite images in the form of the Normalized Difference Vegetation Index (NDVI). The NDVI can be used to identify the density of greenness per unit area but cannot distinguish the types of greenspaces. In addition, due to the characteristics of the data, it was difficult to prepare data that covers a wide area as in this study, for the same period.

To compensate for this disadvantage, a combination of Light Detection and Ranging (LiDAR) data, a three-dimensional laser survey, which enables researchers to distinguish between the types of greenspaces such as trees, shrub, and grass using height data, exists [40]. LiDAR data, with its large amount of data and cost of imaging, are more suited for comparing small cities, but it is difficult to use for large-scale surveys targeting dozens of municipalities, such as in this study. Therefore, in our study, the types of greenspaces were evaluated using the JAXA data that classified the types of greenspaces from satellite images, in response to a large-scale survey. Secondly, our study differs from prior work based on the analyzed regional units. Previous studies evaluated the greenspaces in buffers from addresses used as representative points [8,9,41,42]. The buffers used here are often in the range of 300 to 3000 m. Thus, the greenspace was analyzed previously at an individual level. In this study, in order to evaluate the impact at the neighborhood level, we conducted a multi-level analysis of school districts. This study showed similar results with those of previous studies, despite the differences outlined above.

### 4.4. Limitations

Several limitations of this study should be mentioned. First, this is a cross-sectional study which cannot reveal the effect of reverse causation. Secondly, as the data is based on a questionnaire, recall bias may have occurred. Thirdly is the calculation of the amount of greenspace. The data used in this report indicate the types of greenspaces for each mesh but not the density of the greenness, unlike that of the NDVI. Moreover, the amount of greenspace exposure according to each person’s behavior was not clear. In the future, studies considering these limitations will be required, using quantitative data, such as NDVI and GPS. Furthermore, it is necessary to analyze the amount of greenspace, including trees, occurring at the eye level on the basis of the local characteristics (e.g., urban/rural) and their appearance as natural sights in order to improve the physical environment and develop new architecture and city plans.

## 5. Conclusions

In conclusion, we clarified that the types and amounts of greenspaces showed different associations with the degree of depression in older adults in Japan depending on the regional (whether urban or rural) characteristics. Lower odds of depression were associated with a large amount of greenspace among Japanese older adults. Urban areas with an abundance of trees and rural areas with moderate amounts of grassland were associated with lower odds of depression.

In the future, our findings could be effective as guidelines for primordial prevention through the development of planned greenspaces according to the regional characteristics. These findings may be useful in policymaking for urban and architectural planners to determine the need for secure greenspaces, as well as the types of greenspaces that should be maintained in urban and rural areas.

## Figures and Tables

**Table 1:** Participants’ descriptive statistics.

Variables	Total (*n* = 126,878)		Depression (*n* = 25,846; 20.4%)		No Depression (*n* = 101,032; 79.6%)	
	Number	%	Number	%	Number	%
Individual level variables						
Sex						
Men	61,493	48.5	12,880	49.8	48,613	48.1
Women	65,385	51.5	12,966	50.2	52,419	51.9
Age (years)						
65–69	42,150	33.2	8379	32.4	33,771	33.4
70–74	35,398	27.9	6880	26.6	28,518	28.2
75–79	27,928	22.0	5678	22.0	22,250	22.0
80–84	15,058	11.9	3377	13.1	11,681	11.6
≥85	6344	5.0	1532	5.9	4812	4.8
Educational attainment (years)						
<10	37,736	29.7	9469	36.6	28,267	28.0
≥10	87,866	69.3	16,056	62.1	71,810	71.1
Missing	1276	1.0	321	1.2	955	0.9
Annual household income (Dollars)						
<20,000	48,843	38.5	12,518	48.4	36,325	36.0
20,000–39,999	42,306	33.3	6502	25.2	35,804	35.4
≥40,000	12,174	9.6	1231	4.8	10,943	10.8
Missing	23,555	18.6	5595	21.6	17,960	17.8
Living with others						
No (living alone)	17,802	14.0	5273	20.4	12,529	12.4
Yes	89,969	70.9	16,675	64.5	73,294	72.5
Others/Missing	19,107	15.1	3898	15.1	15,209	15.1
Employment situation						
Working	33,158	26.1	5487	21.2	27,671	27.4
Retired and not working now	69,091	54.5	14,603	56.5	54,488	53.9
Never had a job	7590	6.0	1693	6.6	5897	5.8
Missing	17,039	13.4	4063	15.7	12,976	12.8
Frequency of going outside (per week)						
≥4 times	95,059	74.9	16,374	63.4	78,685	77.9
<4 times	30,715	24.2	9210	35.6	21,505	21.3
Missing	1104	0.9	262	1.0	842	0.8
Drive a car						
No	52,358	41.3	12,421	48.1	39,937	39.5
Yes	74,520	58.7	13,425	51.9	61,095	60.5
The longest type of occupation						
Other than AG*	102,857	81.1	20,489	79.3	82,368	81.5
AG*	4396	3.5	985	3.8	3411	3.4
Never had a job	7192	5.7	1647	6.4	5545	5.5
Missing	12,433	9.8	2725	10.5	9708	9.6
Residence years						
<10 years	10,794	8.5	2987	11.6	7807	7.7
≥10 years	114,637	90.4	22,452	86.9	92,185	91.2
Missing	1447	1.1	407	1.6	1040	1.0

AG*: agriculture, forestry and fisheries. Annual household income was reported in Japanese Yen and converted at a rate of 100 US Dollars per Yen.

**Table 2:** Summary of neighborhood measures and their Spearman associations (*n* = 881).

Neighborhood Measures	Mean	SD	Median	Min	Max	1	2	3	4
**Total (*n* = 881)**									
1. All kinds of green field (area ratio)	37.3	32.2	1.1	0.0	99.8	1.000			
2. Trees (area ratio)	15.5	22.7	0.8	0.0	98.7	**0.781** **	1.000		
3. Fields (area ratio)	20.1	20.6	0.7	0.0	91.6	**0.766** **	**0.389** **	1.000	
4. Grasslands (area ratio)	1.7	3.1	0.1	0.0	39.7	**0.696** **	**0.510** **	**0.520** **	1.000
5. Residential population density (population/km^2^)	9550.9	5166.6	174.1	795.8	37915.6	**−0.688** **	**−0.345** **	**−0.623** **	**−0.537** **

** *p* < 0.01. SD, standard deviation; Min, minimum; max, maximum.

**Table 3:** Odds ratios (ORs) with 95% confidence intervals (95% CI) for the association of greenspace and depression among older Japanese adults (*n* = 126,878).

Variables	Number	Model 1		Model 2	
		OR (95% CI)		OR (95% CI)	
**All types of greenspaces (area ratio)**					
Lowest tertile (0–25.66)	42,500	1.00			
Middle tertile (25.66–70.86)	41,910	0.97	(0.93–1.02)		
Highest tertile (≥70.86)	42,468	**0.90**	**(0.85–0.95)** *		
**Trees (area ratio)**					
Lowest tertile (0–2.00)	42,341			1.00	
Middle tertile (2.00–16.96)	42,230			**0.92**	**(0.88–0.96)** *
Highest tertile (16.97–98.69)	42,307			**0.93**	**(0.88–0.99)** *
**Grasslands (area ratio)**					
Lowest tertile (0–0.25)	42,266			1.00	
Middle tertile (0.25–2.06)	42,231			0.98	(0.93–1.03)
Highest tertile (2.07–39.7)	42,381			0.97	(0.92–1.03)
**Fields (area ratio)**					
Lowest tertile (<13.38)	42,340			1.00	
Middle tertile (13.38–36.11)	42,494			1.01	(0.96–1.06)
Highest tertile (≥36.12)	42,044			1.01	(0.95–1.07)
**Residential population density (persons per kilometer squared)**					
Lowest tertile (795.6–4508.5)	42,256	1.00		1.00	
Middle tertile (4508.5–8957.5)	42,286	**0.94**	**(0.89–1.00)** *	0.97	(0.92–1.02)
Highest tertile (8957.5–37,915.6)	42,336	**0.89**	**(0.84–0.95)** *	**0.94**	**(0.88–1.00)** *

* *p* < 0.05. The random-effect estimate (standard error) of the urban areas’ null model was 0.03 (0.001). Unknown data of variables in the dataset were categorized as missing in this analysis. All the models were adjusted for individual-level variables (sex, age, educational attainment, annual household income, living with others, employment situation, frequency of going outside, driving a car, residence duration in years, the longest type of occupation) and neighborhood-level variables (annual total daylight, deepest annual snowfall amount, annual rainfall, residential population density).

**Table 4:** Odds ratios (ORs) with 95% confidence intervals (95% CIs) for the association of greenspace and depression among older Japanese adults.

Variables	Urban (*n* = 93,055)		Rural (*n* = 33,823)	
	Model 1	Model 2	Model 1	Model 2
	OR (95% CI)	OR (95% CI)	OR (95% CI)	OR (95% CI)
**All types of greenspaces (area ratio)**				
Lowest tertile	1.00		1.00	
Middle tertile	0.97		0.97	
	(0.93–1.02)		(0.88–1.07)	
Highest tertile	0.96		0.94	
	(0.91–1.00)		(0.85–1.03)	
**Trees (area ratio)**				
Lowest tertile		1.00		1.00
Middle tertile		**0.95**		0.93
		**(0.90–1.00)** *		(0.84–1.02)
Highest tertile		**0.94**		1.00
		**(0.89–1.00)** *		(0.89–1.13)
**Grasslands (area ratio)**				
Lowest tertile		1.00		1.00
Middle tertile		1.01		**0.91**
		(0.96–1.06)		**(0.83–1.00)** *
Highest tertile		1.00		0.92
		(0.94–1.06)		(0.82–1.02)
**Fields (area ratio)**				
Lowest tertile		1.00		1.00
Middle tertile		1.02		1.06
		(0.97–1.08)		(0.95–1.17)
Highest tertile		1.03		1.14
		(0.96–1.09)		(1.01–1.28)

* *p* < 0.05. The random-effect estimates (standard error) of the urban and rural areas’ null models were 0.03 (0.01) and 0.02 (0.01), respectively. All the models were adjusted for individual-level variables (sex, age, educational attainment, annual household income, living with others, employment situation, frequency of going outside, driving a car, residence duration in years, the longest type of occupation), and neighborhood-level variables (total daylight hours, deepest annual snowfall amount, annual rainfall). Unknown data of variables in the dataset were categorized as missing in this analysis. The cutoff (tertiles 1 to 3) of urban’s greenspace tertiles for all types of greenspaces (<18.1, 18.1–52.8, and ≥52.8), trees (<1.5, 1.5–11.9, and ≥11.9), grasslands (<0.1, 0.1–1.4, and ≥1.4), and fields (<13.4, 13.4–36.1, and ≥36.1) are shown. The cutoffs (tertiles, lowest to highest) for the rural areas’ greenspace tertiles for all types of greenspaces (<71.0, 71.0–92.6, and ≥92.6), trees (<6.0, 6.0–55.4, and ≥55.4), grasslands (<1.1, 1.1–4.8, and ≥4.8), and fields (<13.4, 13.4–36.1, and ≥36.1) are shown. The numbers (tertiles lowest to highest) for urban areas’ greenspace tertiles for all types of greenspaces (11,189, 11,818, and 10,816), trees (11,289, 11,199, and 11,335), grasslands (11,364, 11,230, and 11,229), and fields (6126, 11,106, and 16,591) are shown. The numbers (tertiles, lowest to highest) for rural areas’ greenspace tertiles for all kinds of greenspaces (29,821, 32,396, and 30,838), trees (30,630, 31,437, and 30,988), grasslands (31,015, 31,275, and 30,765), and fields (36,214, 31,388, and 25,453) are shown.

## References

[1] Fiske A., Wetherell J.L., Gatz M. (2009). Depression in older adults. Annu. Rev. Clin. Psychol..

[2] Pun V.C., Manjourides J., Suh H.H. (2018). Association of neighborhood greenness with self-perceived stress, depression and anxiety symptoms in older U.S adults. Environ. Health.

[3] Nieuwenhuijsen M.J., Khreis H., Triguero-Mas M., Gascon M., Dadvand P. (2017). Fifty shades of green: Pathway to healthy urban living. Epidemiology.

[4] Banay R.F., James P., Hart J.E., Kubzansky L.D., Spiegelman D., Okereke O.I., Spengler J.D., Laden F. (2019). Greenness and depression incidence among older women. Environ. Health Perspect..

[5] Wendelboe-Nelson C., Kelly S., Kennedy M., Cherrie J.W. (2009). A scoping review mapping research on green space and associated mental health benefits. Int. J. Environ. Res. Public Health.

[6] Perrino T., Lombard J., Rundek T., Wang K., Dong C., Gutierrez C.M., Toro M., Byrne M.M., Nardi M.I., Kardys J. (2019). Neighbourhood greenness and depression among older adults. Br. J. Psychiatry.

[7] Taylor L., Hochuli D.F. (2017). Defining greenspace: Multiple uses across multiple disciplines. Landsc. Urban Plan..

[8] Reid C.E., Clougherty J.E., Shmool J.L., Kubzansky L.D. (2017). Is all urban green space the same? A comparison of the health benefits of trees and grass in New York City. Int. J. Environ. Res. Public Health.

[9] Sarkar C., Webster C., Pryor M., Tang D., Melbourne S., Zhang X., Jianzheng L. (2015). Exploring associations between urban green, street design and walking: Results from the Greater London boroughs. Landsc. Urban Plan..

[10] Browning M.H.E., Lee K., Wolf K.L. (2019). Tree cover shows an inverse relationship with depressive symptoms in elderly residents living in U.S. nursing homes. Urban For. Urban Green..

[11] Alcock I., White M.P., Lovell R., Higgins S.L., Osborne N.J., Husk K., Wheeler B.W. (2015). What accounts for ‘England’s green and pleasant land’? A panel data analysis of mental health and land cover types in rural England. Landsc. Urban Plan..

[12] Wahl H.W. (2005). Ageing research along the urban-rural distinction: Old questions and new potential. Eur. J. Ageing.

[13] Marcellini F., Giuli C., Gagliardi C., Papa R. (2007). Aging in Italy: Urban-rural differences. Arch. Gerontol. Geriatr..

[14] Dong X.Q., Simon M.A. (2010). Health and aging in a Chinese population: Urban and rural disparities. Geriatr. Gerontol. Int..

[15] Kato K., Kondo K., Takeda T. (2015). Are there inter-municipality differences in the ratio of individuals with limited activities of daily living?: The JAGES Project. Jpn. Occup. Ther. Res..

[16] Ide K., Tsuji T., Kanamori S., Jeong S., Nagamine Y., Kondo K. (2020). Social participation and functional decline: A comparative study of rural and urban older people, using Japan Gerontological Evaluation Study longitudinal data. Int. J. Environ. Res. Public Health.

[17] Kondo K. (2016). Progress in aging epidemiology in Japan: The JAGES project. J. Epidemiol..

[18] Kondo K., Rosenberg M. (2018). Advancing Universal Health Coverage through Knowledge Translation for Healthy Ageing: Lessons Learnt from the Japan Gerontological Evaluation Study.

[19] Fujiwara T., Takamoto I., Amemiya A., Hanazato M., Suzuki N., Nagamine Y., Sasaki Y., Tani Y., Yazawa A., Inoue Y. (2017). Is a hilly neighborhood environment associated with diabetes mellitus among older people? Results from the JAGES 2010 Study. Soc. Sci. Med..

[20] Sheikh J.I., Yasavage J.A. (1986). Geriatric Depression Scale (GDS) Recent evidence and development of a shorter version. Clin. Gerontol..

[21] Rinaldi P., Mecocci P., Benedetti C., Ercolani S., Bregnocchi M., Menculini G., Catani M., Senin U., Cherubini A. (2003). Validation of the five-item Geriatric Depression Scale in elderly subjects in three different settings. J. Am. Geriatr. Soc..

[22] Tani Y., Sasaki Y., Haseda M., Kondo K., Kondo N. (2015). Eating alone and depression in older men and women by cohabitation status: The JAGES longitudinal survey. Age Ageing.

[23] Murata C., Kondo K., Hirai H., Ichida Y., Ojima T. (2008). Association between depression and socio-economic status among community-dwelling elderly in Japan: The Aichi Gerontological Evaluation Study (AGES). Health Place.

[24] Alos H.P. ALOS-2/PALSAR-2 Observation Result for Eruption of Mt. Fuego in Guatemala. https://www.eorc.jaxa.jp/ALOS/en/index.htm.

[25] Takahashi M., Nasahara N.K., Tadono T., Watanabe T., Dotsu M., Sugimura T., Tomiyama N. Jaxa High Resolution Land-Use and Land-Cover Map of Japan. Proceedings of the IEEE International Symposium on Geoscience and Remote Sensing (IGARSS).

[26] Kanamori S., Takamiya T., Inoue S., Kai Y., Tsuji T., Kondo K. (2018). Frequency and pattern of exercise and depression after two years in older Japanese adults: The JAGES longitudinal study. Sci. Rep..

[27] Kasof J. (2009). Cultural variation in seasonal depression: Cross-national differences in winter versus summer patterns of seasonal affective disorder. J. Affect. Disord..

[28] Witham M.D., Donnan P.T., Vadiveloo T., Sniehotta F.F., Crombie I.K., Feng Z., McMurdo M.E. (2014). Association of day length and weather conditions with physical activity levels in older community dwelling people. PLoS ONE.

[29] Meteorological Data of National Land Information. http://nlftp.mlit.go.jp/ksj/gml/datalist/KsjTmplt-G02.html.

[30] Land Use Data of National Land Information. http://nlftp.mlit.go.jp/ksj/gml/datalist/KsjTmplt-L03-b.html.

[31] FUA Functional Urban Area. https://www.oecd.org/cfe/regionaldevelopment/Japan.pdf.

[32] Cabinet Office Reiwa 1st Year White Paper on Aging Society (Overall Version). https://www8.cao.go.jp/kourei/whitepaper/w-2019/zenbun/01pdf_index.html.

[33] Helbich M., Yao Y., Liu Y., Zhang J., Liu P., Wang R. (2019). Using deep learning to examine street view green and blue spaces and their associations with geriatric depression in Beijing, China. Environ. Int..

[34] Stevenson M.P., Schilhab T., Bentsen P. (2018). Attention Restoration Theory II: A systematic review to clarify attention processes affected by exposure to natural environments. J. Toxicol. Environ. Health B Crit. Rev..

[35] Kaplan R., Kaplan S. (1989). The Experience of Nature: A Psychological Perspective.

[36] Rajoo K.S., Karam D.S., Abdullah M.Z. (2020). The physiological and psychosocial effects of forest therapy: A systematic review. Urban For. Urban Green..

[37] Morita E., Fukuda S., Nagano J., Hamajima N., Yamamoto H., Iwai Y., Nakashima T., Ohira H., Shirakawa T.J. (2007). Psychological effects of forest environments on healthy adults: Shinrin–yoku (forest–air bathing, walking) as a possible method of stress reduction. Public Health.

[38] Maas J., Verheij R.A., Groenewegen P.P., De Vries S., Spreeuwenberg P. (2006). Green space, urbanity, and health: How strong is the relation?. J. Epidemiol. Community Health.

[39] Jiang B., Chang C.Y., Sullivan W.C. (2014). A dose of nature: Tree cover, stress reduction, and gender differences. Landsc. Urban Plan..

[40] Casalegno S., Anderson K., Cox D.T.C., Hancock S., Gaston K.J. (2017). Ecological connectivity in the three-dimensional urban green volume using waveform airborne lidar. Sci. Rep..

[41] Gascon M., Sánchez-Benavides G., Dadvand P., Martínez D., Gramunt N., Gotsens X., Cirach M., Vert C., Molinuevo J.L., Crous-Bou M. (2018). Long-term exposure to residential green and blue spaces and anxiety and depression in adults: A cross-sectional study. Environ. Res..

[42] Brown S.C., Perrino T., Lombard J., Wang K., Toro M., Rundek T., Gutierrez C.M., Dong C., Plater-Zyberk E., Nardi M.I. (2018). Health disparities in the relationship of neighborhood greenness to mental health outcomes in 249,405 U.S. Medicare beneficiaries. Int. J. Environ. Res. Public Health.

